# An electrophysiological investigation on the emotion regulatory mechanisms of brief open monitoring meditation in novice non-meditators

**DOI:** 10.1038/s41598-020-71122-7

**Published:** 2020-08-28

**Authors:** Yanli Lin, Lilianne M. Gloe, Courtney C. Louis, William D. Eckerle, Megan E. Fisher, Jason S. Moser

**Affiliations:** 1 Department of Psychology, Michigan State University, Psychology Building, 69-E, East Lansing, MI USA; 2 Department of Psychology, The Ohio State University, Columbus, OH USA

**Keywords:** Psychology, Emotion

## Abstract

Despite a growing literature supporting the salutary effects of mindfulness meditation on emotion regulation, the underlying mechanisms linking neural and subjective changes occurring *during* the actual practice of meditation with emotion regulatory effects observed *after* meditation remains virtually unexplored. The current study sought to address this gap in knowledge by testing the hypothesis that adoption of internally-directed focused attention, indexed by increased alpha and theta spectral power, during brief open monitoring (OM) mindfulness meditation predicts reduced emotional reactivity, as measured by the late positive potential (LPP). Results revealed that the OM meditation did not produce demonstrable differences in alpha and theta power but did increase self-reported sleepiness relative to controls. Follow-up analyses showed that sleepiness uniquely moderated the effect of meditation on the LPP, such that less sleepiness during meditation, but not the control audio, corresponded to smaller LPPs to negative images. Change in theta, but not alpha power, between meditation and rest was positively correlated with the LPP even after controlling for sleepiness. Although the primary hypothesis was unsupported, the findings demonstrate that phenomenological and neural changes occurring during OM meditation may modulate its subsequent “off-the-cushion” effects on emotional reactivity.

## Introduction

Originating from a 2,500-year old Buddhist contemplative tradition, mindfulness has received increased interest from people around the world. Although definitions of mindfulness vary across time and context^1^, one of the most cited contemporary definitions of mindfulness refers to the adoption of a nonelaborative, nonjudgmental awareness to present-moment experience^2,3^. Driving its rising popularity, a rapidly growing body of research has shown that adoption and training of mindfulness, and possessing higher dispositional levels of mindfulness, are related to a wide array of benefits^2–5^.

A frequently cited benefit of mindfulness involves its salutary effects on emotion regulation^6,7^, a core self-regulatory ability involving modulation of the generation, experience, and expression of emotion^8^. Despite lay and scientific consensus that mindfulness promotes healthy emotion regulation, little is known about *how* mindfulness confers its emotion regulatory effects. Research into this question is complicated by three factors. First, emotion regulation is conceptualized as a complex dynamic process that unfolds over time^9,10^. Consequently, mechanistic investigations of mindfulness-based emotion regulation may strongly benefit from employing methodologies with temporal sensitivity. Second, mindfulness is a multi-faceted construct differentiable as a state, trait, and mind–body training modality^11,12^. Moreover, mindfulness as mind–body training is itself highly varied, ranging from traditional sitting meditations to a panoply of didactic exercises and integrative interventions^13,14^. This construct heterogeneity complicates operationalization and challenges the ability to draw meaningful inferences from experimental designs (e.g., discriminating the effects of state mindfulness from meditation). Third, one of the most perplexing and obvious challenges in mindfulness research involves understanding how mindfulness training via meditative practice—arguably the most common form of mindfulness training—produces brain changes that underlie emotional well-being. Despite its importance, surprisingly few studies have systematically measured and tested how neural changes occurring during mindfulness meditation relate to “off-the-cushion” emotion regulation. The purpose of the current study was to address these challenges by elucidating a plausible mechanism that links meditative neural activity with emotional reactivity during a subsequent picture viewing task.

### The effects of mindfulness meditation on emotion regulation

The intersection between mindfulness meditation and emotion regulation has long been an area of considerable interest. Both traditional Buddhist descriptions^15^ and more recent theoretical accounts^7,16^ suggest that mindfulness practice may confer emotional benefits. Specifically, the training of present-centered attention and nonjudgmental acceptance of perceived experience has been posited to be foundational towards the development of well-established emotion regulatory skills such as metacognitive awareness^17,18^, narrative self-deidentification^19^, voluntary exposure^20^, and distress tolerance^21^. Given that emotion dysregulation is a prototypic transdiagnostic feature of psychopathology, systematic investigations evaluating the effects and underlying mechanisms of mindfulness meditation on emotional regulation hold significant clinical utility and public health relevance.

Indeed, meta-analytic studies support the emotion regulatory effects of mindfulness meditation in reducing negative emotionality, anxiety, and neuroticism^22^. Longitudinal mindfulness-based interventions (MBIs), which combine didactic instruction with mindfulness meditation, have been shown to improve emotional well-being across samples ranging from healthy students to clinical patients^23^. Experimental studies have also demonstrated that both brief and extended mindfulness meditation practice: (1) lowers the intensity and frequency of negative affect in response to negative situations^24^ and aversive stimuli^25,26^; (2) decreases self-perceived difficulty in regulating emotions^27^; and (3) reduces cognitive interference and autonomic reactivity to emotional stimuli^28^. Further, trait mindfulness has been shown to be robustly correlated with measures of emotional well-being^3^. Given that emotion regulation is an essential feature of mental health and normative functioning^29^, delineating the means through which mindfulness confers its benefits to emotion regulation is crucial for identifying novel therapeutic targets, streamlining effective interventions, and understanding the mind-brain relationship more broadly.

Research aimed at discerning the neural mechanisms of mindfulness-based emotion regulation has predominantly involved neuroimaging studies designed to identify pre-post changes in emotion processing brain activation patterns as a function of mindfulness training. Multiple studies have associated mindfulness training with increased prefrontal activation and reduced activation of the amygdala in response to emotional stimuli^30–33^. Consequently, a popular working hypothesis is that mindfulness training promotes emotion regulation via strengthening prefrontal cognitive control mechanisms that down-regulate affective processing regions^34,35^. Interestingly, this frontal-limbic activation pattern shares significant overlap with the neural correlates of cognitive reappraisal, an emotion regulatory strategy involving semantic reinterpretation of emotional stimuli^36–38^. Such similarities challenge theoretical models that clearly differentiate mindfulness-based emotion regulation (observation and acceptance of emotions without control or action) from cognitive “top-down” regulation strategies (antecedent-focused voluntary manipulation of affective input)^6,7,15^.

Adding further complexity, empirical support for the prefrontal control hypothesis has been equivocal. Holzel and colleagues found an unexpected shift in the relationship between prefrontal and limbic activation^39^. Rather than the increased prefrontal activity corresponding to deceased limbic activity typically observed during top-down emotion regulation, Holzel et al.^39^ reported decreased prefrontal *and* limbic activity in participants after mindfulness training. Such activation patterns introduce the possibility that mindfulness training may promote implicit (i.e., non-voluntary) emotion regulatory processes rather than voluntary down regulation. Moreover, activation of frontal-limbic regions is not reliably detected and has been reported more often in samples of beginning meditators relative to experienced meditators^40^. These inconsistencies reflect two key limitations in this line of work: (1) that voluntary engagement of state mindfulness as an active form of emotion regulation and mindfulness meditation as a practice that implicitly attenuates emotional reactivity may constitute *separate but interrelated processes* within the broader context of mindfulness training; (2) that although changes in regional brain activity have been associated with mindfulness training, little can be inferred about how such changes pertain to the actual *process* of emotion regulation.

### The significance of temporality

Indeed, the inability to discern differences between mindfulness-based emotion regulation and top-down regulation strategies at the neural spatial level has led some researchers to posit that meaningful insights may instead lie in the temporal domain. One promising method to explore this possibility is through using event-related potentials (ERPs), electrical scalp signals that measure stimulus-locked neural activity with millisecond precision. Specifically, the late positive potential (LPP), a central-parietally maximal positive deflection that reaches peak amplitude approximately 300–800 ms after the onset of emotional stimuli, is a well-studied visually evoked ERP measure of emotional processing that has been employed in a variety of emotion regulation studies^41,42^. The LPP is broadly thought to index the motivational significance of visual stimuli such that its amplitude increases with the level of emotional arousal^35–38^. Furthermore, source localization efforts have linked the LPP to areas in the prefrontal cortex, insula, and amygdala, brain structures of emotion processing that have consistently been implicated in studies of mindfulness and emotion^43^. Early time windows (300–1,000 ms) of the LPP are considered to reflect bottom-up attention allocation^36^, whereas later time windows (> 1,000 ms) have been demonstrated to index semantic processing and meaning making^44,45^. Importantly, both time windows appear sensitive to different emotion regulation strategies^46–48^, and have been shown to correlate with self-reported changes in emotional arousal^49^.

Recent studies involving the LPP have yielded promising insights into the emotion regulatory properties of mindfulness. Mindfulness has been broadly associated with reduced LPP responses to negative emotionally evocative stimuli^50–52^. Sobolewski et al. employed a cross sectional design comparing experienced meditators to non-meditating controls, finding smaller LPPs elicited by negative stimuli in mediators relative to controls^50^. Complimenting these findings, Brown et al.^51^ found that higher dispositional mindfulness corresponded to smaller LPP responses to both negative and positive high arousing stimuli, suggesting that trait mindfulness attenuates broadband emotion processing of motivationally salient stimuli^51^. In an experimental study comparing brief open monitoring (OM) mindfulness meditation^6^ with an active control, Lin et al.^52^ found that meditation prior to an affective picture viewing task produced a linear reduction in the difference between negative and neutral LPPs across time^52^. Consequently, Lin and colleagues’ findings suggested that brief mindfulness meditation may reduce emotional reactivity—an integral component of emotion regulation commonly operationalized as the difference in responsivity between negative and neutral trials over time^53,54^. Further, the observed attenuation of the LPP in meditating participants mirrored that of control participants with high dispositional mindfulness, suggesting that the emotion regulatory effects associated with trait mindfulness can be acquired through meditative practice. Critically, these findings converge to support the notion that both prolonged^50^ and brief^52^ mindfulness meditation can engender trait-like attenuations to emotional processing occurring on the order of milliseconds^32,51,55^.

### Operationalizing the multiple facets of mindfulness

Collectively, these studies underscore the importance of approaching mindfulness as a multi-faceted construct^11^, showing that long-term meditative experience, brief meditation practice, and high trait mindfulness are all associated with reduced emotional reactivity. In particular, the distinction between mindfulness as a meditative practice and as an inducible state of mind is often unaccounted for in mindfulness-based emotion regulation studies—obfuscating the extent to which detected effects are attributable to meditation training or on-task engagement of state mindfulness. Exemplifying the utility of experimental ERP designs to answer prevailing questions about mindful emotion regulation, Lin et al.^52^ differentially operationalized mindfulness meditation and state mindfulness, finding that voluntary engagement in state mindfulness (i.e., instructing participants to view the emotional stimuli mindfully) did not produce demonstrable changes in emotional reactivity, nor did it moderate the effects of brief meditation^52^. Instead, as previously mentioned, it was the practice of meditation itself, that lead to subsequent decreases in emotional reactivity. In conjunction with Desbordes’ et al.^32^ observation that participants assuming an ordinary non-mindful state after meditative training exhibited reduced amygdala activity to emotionally aversive stimuli^32^, these findings show that meditation, but not necessarily voluntary engagement of state mindfulness, attenuated emotional reactivity. Indeed, mindfulness meditation in novice non-meditators appears to confer implicit emotion regulatory effects and does not seem to promote explicit emotion regulation involving voluntary antecedent- or response-focused strategies^56^. A critical implication of these findings is to shift investigative attention toward the role of meditative practice—specifically to the link between the neural processes that occur during mediation and the observed changes in emotion processing. In other words, it may be fruitful to extend the aforementioned line of electrophysiological research by examining the extent to which meditative neural changes relate to the emotion regulatory effects (i.e., reduced emotional reactivity) observed after meditation.

### The unique role of meditative practice

Multiple conceptual process models have theorized that the development of internally-directed nonreactive awareness during mindfulness meditation engenders its well-documented benefits to emotional well-being^11,33,34^. However, rigorous testing of these models is limited by the extent that psychological states can be measured during meditation. One potential solution involves measuring EEG neural oscillations—electrical scalp activity that occur at varying rhythmic frequencies. Neural oscillatory activity within specific ranges of frequency (i.e., frequency bands) have been shown to reliably correspond to a variety of psychological states and processes^48,49^. Importantly, a substantive line of research aimed at exploring neural oscillations during mindfulness meditation have detected increased activity in the alpha (8–13 Hz) and theta (4–8 Hz) frequency range. Critically, increased alpha and theta activity (also referred to as increased alpha and theta power) during meditation have been collectively thought to index internally-directed focused attention^57,58^.

Although the functional significance of alpha oscillations has been subject to debate, one leading hypothesis implicates enhanced alpha power in the engagement of internally-directed attention^59–61^. Supporting this view, alpha power has been detected in non-meditative tasks requiring memory, imagination, mental imagery, and inhibition of external stimulation^59,62–64^. Theta power is widely considered a marker of executive attention, as increases in theta power have been detected during cognitive tasks involving sustained attention^65^, attention switching and orientation^66^, memory encoding^67^, and performance monitoring^68^.

Interestingly, the concurrent presence of enhanced alpha and theta power during mindfulness meditation parallels the Theravada Buddhist perspective that development of concentrated internally-directed attention is vital to cultivating the cognitive and emotional benefits of meditation^69^. However, surprisingly few studies have sought to relate meditative neural activity (e.g., alpha and theta power) to dependent measures of emotion processing (e.g., self-reported emotional intensity, LPP, etc.)—creating a “black box” on what might be a *fundamental* level of analysis toward understanding the emotion regulatory properties of mindfulness meditation.

The purpose of the current study was therefore to elucidate a plausible mechanism linking neural oscillatory activity during mindfulness meditation with “off-the-cushion” changes in emotional reactivity. Adapting the experimental procedures described in Lin et al.^52^, novice non-meditators were randomly assigned to complete a guided audio mindfulness meditation or listen to a control audio (collectively referred to as audio induction) prior to completing an affective picture viewing task^52^. Continuous EEG was recorded to measure alpha and theta power during the audio induction, and the LPP during the subsequent picture viewing task. Heeding the repeated calls to adopt a multimodal neuroscientific approach^11,34,35,70^, self-report measures of task compliance, trait mindfulness, and state affect were collected in tandem with neurophysiological data.

In considering the validity of the study design, it must be acknowledged that completion of a brief guided meditation exercise in novice non-meditators represents at best a “first exposure” to mindfulness training and should not be conflated with a developed meditation practice. Single-session mindfulness manipulations involving novice samples are inherently limited, offering a circumscribed yet valuable glimpse into the psychological and neural underpinnings of novel engagement with mindfulness meditation. Indeed, first exposures to mindfulness meditation very likely differ in both process and effect from established long-term practice, rendering unique reasons to study them. Specifically, with the accelerating global popularity of MBIs, understanding how people respond to mindfulness meditation for the first time may confer considerable practical value and clinical utility insofar as such studies may yield measures that predict receptivity and responsivity to mindfulness interventions.

Moreover, to avoid misnomers and semantic ambiguity, we approach the term “mindfulness meditation” from the purview of contemporary western medicine and psychological science while recognizing its historical Buddhist influence. That is to say, mindfulness meditation is construed as a set of mental training practices that is broadly separable into two distinct practices of OM and focused attention (FA; see Lutz et al.^6^), each of which comprise a core component of modern MBIs^71^. Importantly, the brief mindfulness induction employed in the current study is by no means of the same as Buddhist contemplative training. We refer to our experimental manipulation as a “meditation” to broaden accessibility and maintain semantic coherence with wider public discourse, including other scholars within the increasingly interdisciplinary field of contemplative science.

With these caveats in mind, these measures were used to test the central hypothesis that changes in alpha and theta power during meditation relative to rest, reflecting engagement of internally directed focused attention, relates to reduced online emotional reactivity. It was predicted that: 1) meditation-naïve participants randomized to OM mindfulness meditation would exhibit increased alpha and theta power relative to control condition participants; 2) participants assigned to the meditation condition would exhibit less emotional reactivity indexed by reduced LPPs and self-reported negative affect; and 3) the predicted changes in meditative alpha and theta were expected to correlate with the reductions in the LPP. Confirming these predictions would provide compelling evidence that meditative-induced changes in alpha and theta contribute to reduced emotional reactivity across multiple levels of analysis—thereby delineating a plausible mechanism for the salutary effects of mindfulness meditation on emotion regulation.

## Method

### Participants

Two hundred and twelve female students were recruited from Michigan State University’s Human Participation in Research subject pool for course credit (see power analyses described in the ‘Predictions’ section). An all-female sample was recruited to minimize experimental confounds related to gender, and to replicate the screening criteria of Lin et al.^52^. Importantly, previous studies have demonstrated that relative to men, women exhibit higher arousal and a greater LPP response to negative stimuli^72,73^, employ different emotion regulatory strategies^74^, exhibit larger effects of emotion regulation^75,76^ and possibly respond differently to mindfulness meditation^77–79^. Furthermore, because women are more susceptible to mood and anxiety disorders^80,81^ and are more likely to adopt a meditation practice^82^, recruiting an all-female sample confers unique clinical and practical value.

Prospective participants were screened for history of neurological illness and meditation experience. All participants identified as novices, endorsing no prior meditative experience. Consented participants were randomized to either a meditation (*n* = 106) or control group (*n* = 106) involving separate audio inductions. All participants were naïve to group assignments throughout the entire duration of the experiment. One participant was excluded from all analyses due to having a hairstyle that restricted EEG data collection. The remaining two hundred eleven participants (control: *n* = 106, meditation: *n* = 105) had useable data for at least one task of interest, comprising a final sample that ranged in age from 18 to 28 years old (*M* = 19.20, *SD* = 1.34). The majority of the sample identified as Caucasian/White (79.7%); the remaining participants identified as African American/Black (6.9%), Asian (2%), Latino/Hispanic (3.8%), Bi-Racial/Multi-Racial (2.8%), or Other (1.5%).

To maximize data retention, degrees of freedom varied across analyses based on excluded participants. One participant did not complete the questionnaire battery due to experimenter negligence. Six participants were removed from analyses involving the resting task: five to excessive artifacts (i.e., more than 50% of total segments) and one from loss of the data file. Three participants were removed from analyses involving the audio induction due to excessive artifacts. Nine participants were excluded from analyses involving picture viewing due to excessive artifacts that rendered fewer than 12 trials per valence condition (the minimum number of trials needed to maintain reliability; see Moran et al.^83^). Importantly, analyses involved a minimum of 94 participants per group and a maximum of 104 participants per group, exceeding the minimum sample size needed for adequate power (see ‘Predictions’ section below).

### Procedural overview

**Figure 1:**
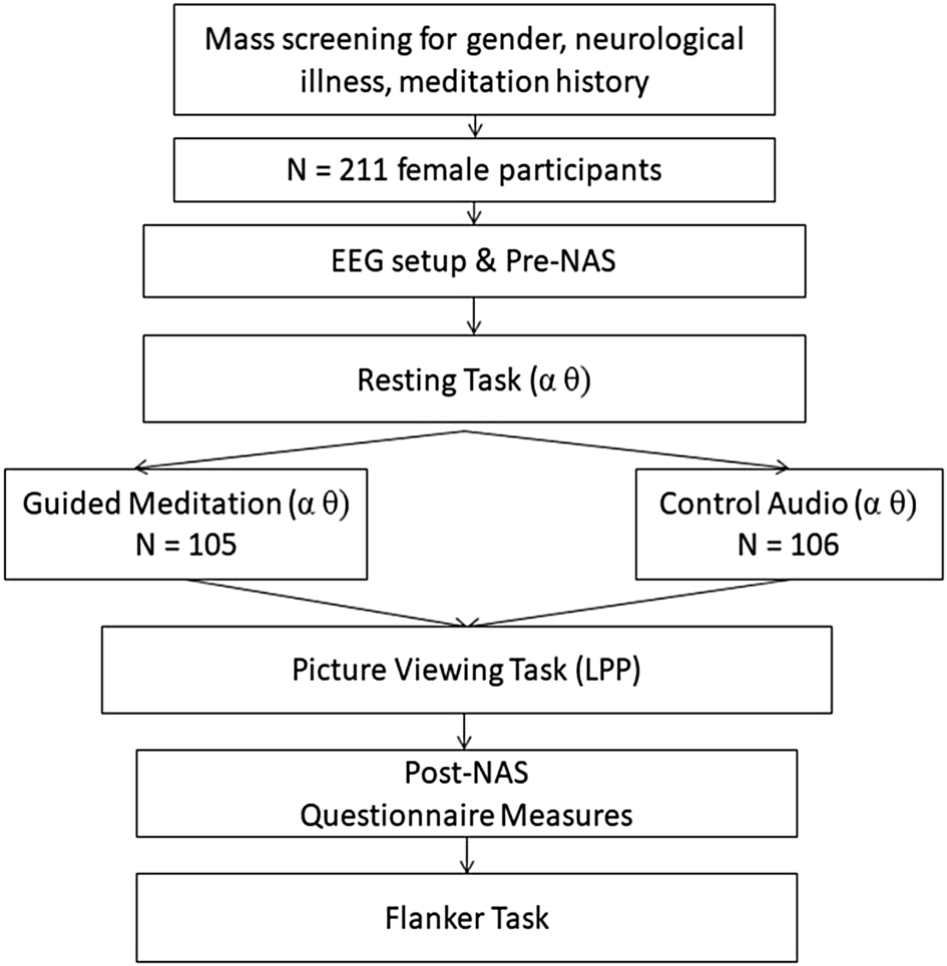
Diagram summarizing participant flow for recruitment, group assignment, and task procedures. *Note*. NAS: negative affect scale; LPP: late positive potential.

The Institutional Review Board at Michigan State University approved all study procedures (IRB #14–871) and all participants provided written informed consent prior to participation. Consequently, the methods described herein were carried out in accordance with the relevant guidelines and regulations. Immediately after consenting, participants completed a brief self-report questionnaire on negative state affect (described in the ‘Measures’ section below). Upon completing the questionnaire, participants were fitted with an electrode cap for EEG recording. Continuous EEG was recorded during completion of three sequential tasks: (1) to measure baseline resting EEG as a means to account for individual differences in non-meditative alpha and theta activity, all participants were instructed to close their eyes and sit quietly for 5 min; (2) participants were then randomly assigned to complete a guided audio meditation exercise or listen to a control audio clip. To control for differences in EEG activity between eyes-closed and eyes-open conditions^84^, participants were instructed to keep their eyes closed during the audio induction; (3) immediately following the induction, participants completed an affective picture viewing task. Upon finishing the task, participants again completed the questionnaire on negative state affect before completing a battery of self-report questionnaires and a manipulation check measure (described in the ‘Measures’ section below). See Fig. 1 for a visual flow diagram of the task procedures. To address a separate research question, participants completed an arrow version of the Eriksen Flanker task to conclude the study visit (these data are reported in Lin et al.^85^).

### Tasks and materials

#### Rest condition

Participants were encouraged to sit relaxed with arms and legs in a comfortable position. Participants were then instructed to close their eyes but not fall asleep and sit quietly after they heard a tone. After 5 min, participants heard the tone again to indicate the end of the task.

#### Audio induction

The meditation induction was comprised of a 20-min guided open monitoring (OM) meditation exercise led by Steve Hickman from the University of San Diego Center for Mindfulness^86^. An OM meditation, as opposed to focused attention (FA) meditation, was selected because of its unique emphasis on fostering nonreactive awareness of arising internal experience—an ability that has been theorized to engender emotion regulatory effects^6,33,87,88^. The recording instructed participants to direct their attention inward, taking notice of present-moment feelings, thoughts, and physical sensations in an open, nonjudgmental manner. Listeners were directed to orient back to their breath when attention wavered.

The control condition involved an 18-min audio recording of a TED talk by the linguist Chris Lonsdale^89^. The recording instructed participants on how to quickly acquire second language fluency. Importantly, the clip was selected to match the duration, didactic style, gender, and speech of the guided meditation.

#### Picture viewing task

Stimuli included 60 pictures taken from the International Affective Picture System (IAPS; Lang et al.^90^). The images were selected and organized into two equal groups on valence and arousal ratings: 30 negative, high arousing pictures and 30 neutral, low arousing pictures. To maximize cross-study generalizability and replicability, the images were identical to the ones presented in Lin et al.^52^. As described previously^52^, the stimuli were presented on a Pentium R Dual Core computer using E-Prime software (Psychology Software Tools, Inc., Sharpsburg, PA, USA) to control the timing and duration of the images. Each image was displayed in color on a 19′’ flat-screen LCD monitor approximately 41′’ from the participant.

On each trial, a white fixation cross ( +) was presented at the center of the screen for 500 ms. A randomly selected image was displayed on the entire screen for 5,000 ms. The inter-trial interval between image offset and fixation onset varied randomly between 2000–4,000 ms. Presentation of the 60 non-repeating images were divided into three blocks of 20 trials, with each block containing 10 negative and 10 neutral images.

### Measures

#### Trait mindfulness

The 39-item Five-Facet Mindfulness Questionnaire (FFMQ)^91^, a psychometrically validated scale that differentiates dispositional mindfulness into five facets, was used to check for baseline differences in trait mindfulness between the experimental groups. Accounting for possible group differences in trait mindfulness is particularly important because high levels of trait mindfulness have been associated with reduced emotional reactivity^51,52^. The five facets include: (a) observing, defined as noticing internal and external experiences; (b) describing, defined as verbalization of internal experiences; (c) acting with awareness, defined as attending to the present moment experience; (d) nonjudging, defined as adopting a non-evaluative perspective toward thoughts and feelings; and (e) nonreactivity, defined as allowing internal experiences to pass without attachment or elaboration. Participants responded to each item using a 5-point Likert scale ranging from 1 (*never* or *rarely true*) to 5 (*very often* or *always true*).

#### Negative state affect

The negative affect subscale (NAS) of the Positive and Negative Affect Schedule (PANAS)^92^ was used to measure state negative affect. Participants responded to each of the 10 items using a 5-point Likert scale ranging from 1 (v*ery slightly or not at all*) to 5 (*very much*), with higher scores indicating more distress and experience of a particular negative emotion. The NAS exhibits strong psychometric properties and correlates with other measures of psychological distress^93^. Participants were instructed to complete the NAS based on how they felt in the present moment. The NAS was completed twice: once at the start of the experiment (pre-NAS) and once immediately following picture viewing (post-NAS).

#### Manipulation check

Directly replicating Lin et al.^52^, a post-session manipulation check questionnaire was used to assess for potential differences in participant engagement and reception to the experimental manipulation and picture viewing task. Participants rated the extent to which they found the audio induction engaging, interesting, and arousing (1 = *not at all*, 7 = *very*). Participants were also asked to indicate their level of comprehension (1 = *did not understand*, 7 = *completely understand*), emotional reaction (1 = *very negative*, 4 = *neutral*, 7 = *very positive*) and whether they learned anything (1 = *very little*, 7 = *very much*). For the picture viewing task, participants rated their overall engagement, interest, arousal (1 = *not at all*, 7 = *very*), and emotional reaction (1 = *very negative*, 4 = *neutral*, 7 = *very positive*). Specific degree of arousal (1 = *not at all*, 7 = *very*) to neutral and negative pictures was also assessed.

#### Sleepiness

Because of the well-established relationship between sleepiness and alpha synchronization, in addition to previous research showing that novice meditators are particularly susceptible to sleepiness and drowsiness during meditation^94^, participants were required to report their sleepiness (1 = *feeling active, vital, alert, or wide awake*, 8 = *I fell asleep*) during the 5-min resting task, audio induction, and picture viewing task separately using the Stanford Sleepiness Scale^95^.

### Electrophysiological recording and data reduction

As described in our previous studies^52,85,96^, continuous EEG was recorded using active Ag/AgCl electrodes (BioSemi ActiveTwo) placed at the left and right mastoids and 64 scalp sites per the modified 10–20 system. Electrodes placed on the left and right mastoids served as a reference—the average activity of the mastoids was subtracted from each scalp site to isolate electrical scalp activity. To remove ocular artifacts from the EEG data, the electrooculogram (EOG) activity generated from eye movement and blinks was recorded from electrodes placed at the outer cathi of each eye, and above (at site FP1) and below the left eye. The common-mode sense active electrode and the driven right-leg passive electrode formed the ground during data acquisition. All signals were digitized at 1,024 Hz.

Offline analyses were performed using Brain Vision Analyzer 2 (BrainProducts, Gilching, Germany). The EEG signals were re-referenced to the average of the left and right mastoids. Ocular artifacts were corrected using the algorithm developed by Gratton et al.^97^. All signals were low-pass filtered at 20 Hz and high-pass filtered at 0.01 Hz. An artifact rejection algorithm was applied to automatically reject trials and segments containing excessive movement, facial muscle activity, sweat, and other physiological artifacts based on the following criteria: a voltage step of more than 50 uV between sample points, a voltage difference of more than 400 uV within 200 ms intervals, voltage exceeding ± 200 uV, and a maximum voltage difference of less than 0.50 uV within 1,000 ms intervals.

### Power spectral analysis

The EEG recorded during rest and the audio induction was partitioned into 2-s epochs. A fast Fourier transform (FFT), used to convert the data from the temporal to frequency domain, was then applied to all artifact-free epochs after the data was weighted with a hamming window that tapers the distal 10% of each epoch. The data was then averaged across epochs and integrated spectral power was computed for the alpha (8–13 Hz) and theta (4–8 Hz) frequency bands. Following the regional division outlined in Lagopoulos et al.^98^, spectral power at each electrode site was averaged across 3 regions of interest (ROIs) across the scalp– frontal (F8, F6, F4, F2, Fz, F1, F3, F5, F7, AF8, AF4, AFz, AF3, AF7, Fp2, Fpz, Fp1, FT8, FC6, FC4, FC2, FCz, FC1, FC3, FC5, FT7), temporal-central (T8, C6, C4, C2, Cz, C1, C3, C5, T7, TP8, CP6, CP4, CP2, CPz, CP1, CP3, CP5, TP7), and posterior (P10, P8, P6, P4, P2, Pz, P1, P3, P5, P7, P9, PO8, PO4, POz, PO3, PO7, O2, Oz, O1, Iz). All values were log transformed to normalize their distribution. Difference values between the audio induction and rest were computed to capture within-subject changes in alpha and theta power (henceforth referred to as meditative alpha and meditative theta, respectively).

### Picture viewing analysis

For the picture viewing task, EEG epochs of 5,200 ms (200 ms baseline) were extracted from the continuous data file for analysis. Consistent with prior work^52,99^, the LPP was partitioned based on early and late time windows in order to examine the effects of the experimental manipulation on early automatic attention and later semantic processing, respectively. Adapting the parameters specified in Lin et al.^52^, the electrophysiological activity during the early window was termed the *early maximal LPP* and quantified as the average voltage across the 500–900 ms time window (± 200 ms from which the LPP was maximally positive [700 ms]). The late window response was termed the *late sustained LPP* and quantified as the average voltage across successive 1,000 ms time windows ranging from 1,000 to 5,000 ms post-stimulus onset. The LPP was calculated at the electrode site Pz, where its amplitude was maximal. Importantly, the data reduction strategy (i.e., baseline, time window, and electrode selection) was determined a priori based on previous work and never altered for re-analysis or exploratory means. Consistent with established guidelines^100^, the rationale driving our approach was to maintain methodological continuity, reduce chance of type 1 error, and enhance cross-study generalizability.

### Predictions

First, we predicted that there would be no baseline group differences in measures of trait mindfulness, negative state affect, and sleepiness across the tasks; participant responses to the manipulation check was expected to replicate Lin et al.^52^. FFMQ, pre-NAS, sleepiness ratings, and manipulation check responses were submitted to independent-samples t-tests with Group (meditation, control) as a between-subject variable. Given that the audio induction and manipulation check items were identical to Lin et al.^52^, manipulation check responses were expected to replicate. Namely, that participants would rate the control audio to be more interesting and endorse learning more relative to the guided meditation. Other ratings on the manipulation check were not expected to differ by group.

Second, we predicted that alpha and theta power would increase during the audio induction relative to rest for meditation participants but not controls. Log-transformed alpha and theta values were subjected to two separate 2 Task (audio, rest) X 3 ROI (frontal, temporal-central, posterior) repeated measures ANOVAs (rANOVAs) with Group (meditation, control) as a between-subject factor. Based on prior research^58^, a significant Task X Group interaction was expected such that both alpha and theta were predicted to uniquely increase during the audio induction from rest in the meditation relative to control group. Because regional differences in meditative alpha and theta vary across studies^98,101–103^, no specific predictions regarding interactions involving ROI were made. Independent-sample t-tests were also used to directly compare the log-transformed band power between the audio inductions (i.e., alpha and theta power during meditation vs. control). It was expected that meditating participants would exhibit greater alpha and theta power relative to non-meditating controls. A power analysis using an aggregate effect size (*d* = 0.93 for alpha, *d* = 1.21 for theta) from previous studies involving novice meditators^102–106^ indicated that a minimum sample of 12 participants was needed to detect the within-subject increase in alpha power between meditation and rest with a power of 0.80. However, because the current study involved an active control group, a power analysis using a conservative effect size estimate of *d* = 0.30 indicated that a minimum sample of 90 total participants was needed to detect the predicted interaction with a power of 0.80.

Third, we predicted that there would be reduced LPP responses and lower ratings of negative state affect in the meditation but not control group. The primary LPP analysis consisted of two rANOVAs. The early maximal LPP was submitted to a 2 Valence (negative, neutral) one-factor rANOVA with Group (meditation, control) as a between-subject factor. The late sustained LPP was submitted to a 2 Valence (negative, neutral) X 4 Time (1,000–2000, 2000–3,000, 3,000–4,000, 4,000–5,000 ms) rANOVA with Group (meditation, control) as a between-subject factor. Main and interactive effects involving Time and Valence were analyzed using within-subject contrasts. Greenhouse–Geisser corrections were applied to *p*-values associated with multiple *df* repeated measures comparisons when appropriate. Based on previous work^52,107^, the manipulation was not expected to modulate the early maximal LPP. However, given that the early maximal LPP is negatively correlated with trait mindfulness and meditation experience^50–52^, it was possible that the increased sample size of the current study could reveal early LPP attenuation in the meditation relative to the control group. Seeking to replicate Lin et al.^52^, it was predicted that there would be a significant Time X Valence X Group interaction, such that the difference in LPP amplitude by valence would decrease linearly over time for the meditation but not for the control group. Similarly, an rANOVA involving Time (pre, post) as the within-subject factor and Group (meditation, control) as a between-subject factor was conducted to test the prediction that the difference between post- and pre-NAS would be smaller in the meditation group relative to controls. A power analysis using the Lin et al.^52^ effect size (*d* = 0.46) indicated that a sample of 48 total participants (24 per group) was needed to detect this effect with a power of 0.80.

Lastly, predicted increases in meditative alpha and theta (prediction 2) were expected to relate to smaller differences between the LPP response to negative and neutral stimuli (prediction 3). Importantly, these relationships were expected to be evidenced in only the meditation but not control group via a Fisher r-to-z test of independent correlations. This would demonstrate that meditation-induced changes in alpha and theta power predict attenuation of emotion reactivity. Due to the novelty of this analysis (to our knowledge, no study has examined relationships between meditative neural oscillatory activity with the LPP), a power analysis using a medium effect size estimate (*q* = 0.37) suggested that a sample of 188 participants (94 per group) was needed to detect the proposed effect with a power of 0.80. Because this analysis required the most participants, a total of 188 participants were selected as the minimum target sample size. Given that previous studies relating alpha power with measures of emotional arousal (self-report arousal^108^; self-report arousal and LPP^109^) yielded an aggregate effect size of *r* = 0.47, the effect estimate was a relatively conservative approximation.

## Results

### Prediction 1: baseline trait mindfulness, NAS, manipulation check, and sleepiness

Descriptive statistics of questionnaire measures and manipulation check responses by group are presented in Tables 1 and 2, respectively. Importantly, there were no group differences in dispositional mindfulness (five factors, overall), or pre-experiment negative affect (*t*s <|1.38|, *p*s > 0.169).

**Table 1:** Means and SD of self-report battery by group.

Variable	Control N = 106			Meditation N = 104		
	*Range*	*M*	*SD*	*Range*	*M*	*SD*
FFMQ overall	86–175	124.83	17.62	86–165	124.15	16.79
FFMQ observe	11–37	25.47	5.37	17–40	26.48	5.22
FFMQ describe	9–36	26.25	5.42	12–38	26.08	5.69
FFMQ acting with awareness	10–39	27.40	5.94	12–39	26.44	5.72
FFMQ nonjudgment	9–38	25.71	7.12	10–38	25.44	6.47
FFMQ nonreactivity	11–34	20.00	4.52	12–30	19.71	3.94
NAS pre-experiment	10–28	11.96	3.25	10–30	11.89	2.77
NAS post-experiment	10–42	15.11	5.42	10–41	16.01	5.96
Sleepiness rest	1–7	3.66	1.41	1–6	3.52	1.45
Sleepiness audio	1–6	3.79	1.42	1–7	4.35	1.48
Sleepiness picture viewing	1–6	1.90	1.09	1–5	1.70	0.92

*FFMQ* five factor mindfulness questionnaire (high scores indicate higher levels of dispositional mindfulness, overall score computed as average of all items); *NAS* negative affect scale (higher scores indicate greater negative state affect).

**Table 2:** Means and SD of manipulation check ratings by group.

Variable	Control N = 106			Meditation N = 104		
	*Range*	*M*	*SD*	*Range*	*M*	*SD*
Audio engagement	1–7	4.23	1.42	1–7	4.18	1.59
Audio interest	1–7	4.54	1.63	1–7	3.56	1.66
Audio emotional reaction	2–7	4.76	1.00	1–7	4.48	1.06
Audio arousal	1–6	3.13	1.53	1–6	2.80	1.52
Audio understanding	1–7	5.37	1.42	1–7	5.44	1.60
Audio learning	1–7	4.65	1.39	1–6	3.52	1.41
Picture viewing engagement	1–7	5.93	1.28	2–7	5.94	1.13
Picture viewing interest	1–7	5.07	1.32	1–7	5.16	1.31
Picture viewing neutral arousal	1–7	2.62	1.62	1–7	2.95	1.52
Picture viewing negative arousal	1–7	5.40	1.94	1–7	5.12	1.94
Picture viewing overall arousal	1–7	4.75	1.65	1–7	4.75	1.72
Picture viewing emotional reaction	1–6	2.90	1.19	1–6	2.78	1.21

Audio emotional reaction (lower scores below 4 indicate more negative emotional response, higher scores above 4 indicate more positive emotional response); Picture viewing emotional reaction ((lower scores below 4 indicate more negative emotional response, higher scores above 4 indicate more positive emotional response).

Replicating Lin et al.^52^, independent-samples t-tests comparing participant responses to the audio recording revealed group differences in interest (*t*(1, 208) = 4.31, *p* < 0.001), and learning (*t*(1, 208) = 5.85, *p* < 0.001), such that participants who listened to the control audio rated the induction as more interesting (controls: *M* = 4.54, *SD* = 1.63, meditation: *M* = 3.56, *SD* = 1.66), and endorsed learning more (controls: *M* = 4.65, *SD* = 1.39, meditation: *M* = 3.52, *SD* = 1.41). The groups also differed slightly in emotional response (*t*(1, 208) = 1.99, *p* = 0.048), such that participants reacted more positively to the control audio (*M* = 4.76, *SD* = 1.00) than the guided meditation (*M* = 4.48, *SD* = 1.01). Importantly, there were no differences in engagement, arousal, or understanding (*t*s <|1.59|, *p*s > 0.113), suggesting that participants approached the inductions with equal levels of engagement and comprehension. Comparing participant responses to the picture viewing task yielded no group differences. Specifically, there were no differences in engagement, interest, arousal, and overall emotional response (*t*s <|1.52|, *p*s > 0.131).

Lastly, comparing participant ratings of sleepiness across tasks yielded an unexpected group difference during only the audio induction (*t*(1, 208) = 2.77, *p* = 0.006), such that participants reported higher levels of sleepiness during the guided meditation (*M* = 4.35, *SD* = 1.48) relative to the control audio (*M* = 3.79, *SD* = 1.42). Sleepiness did not differ between groups during the rest or picture viewing task (*t*s <|1.40|, *p*s > 0.164). This constellation of sleepiness ratings suggested that the guided meditation may have *induced* sleepiness and counters the possibility of a baseline group difference in overall sleepiness.

Importantly, sleepiness has been shown to modulate both alpha and theta activity^110^. Therefore, increased sleepiness during the guided meditation could have confounded alpha and theta power, and potentially obfuscated the testing of both the expected effect (i.e., reduced LPP) and hypothesized neural mechanism. Moreover, a difference in sleepiness between the experimental conditions may provide a compelling alternative explanation to the findings of Lin et al.^52^, such that increased sleepiness induced during the guided meditation, rather than the actual practice of meditation, moderated the LPP. To adequately address these possibilities, mean-centered audio induction sleepiness ratings were entered as a continuous predictor in the proposed rANOVAs (predictions 2 and 3) and partialled as a control variable in the planned correlations between meditative neural oscillatory activity and the LPP (prediction 4). When significant effects of sleepiness ratings were detected, follow-up correlational analyses were conducted to aid in interpretation of results.

### Prediction 2: alpha and theta power during rest and audio induction

Descriptive statistics of alpha and theta values are presented in Table 3. To check for baseline differences in resting state alpha and theta power, log-transformed alpha and theta values across ROI (frontal, temporal-central, posterior) were submitted to a one-way ANOVA with Group as a between-subject factor. As expected, no group differences emerged across any region for alpha or theta power during rest (*F*s < 2.40, *p*s > 0.123).

**Table 3:** Means and SD of log-transformed alpha and theta values as function of task, site, and group.

Variable	Control rest: N = 102, Induction: N = 104			Meditation rest: N = 103, Induction: N = 104		
	*Range*	*M*	*SD*	*Range*	*M*	*SD*
Rest alpha frontal	0.18–1.06	0.57	0.21	0.17–1.42	0.61	0.24
Rest alpha temporal-central	0.18–1.26	0.64	0.24	0.17–1.41	0.68	0.25
Rest alpha posterior	0.24–1.61	0.85	0.32	0.18–1.58	0.89	0.31
Induction alpha frontal	0.18–1.14	0.55	0.21	0.15–1.41	0.58	0.23
Induction alpha temporal-central	0.14–1.25	0.60	0.24	0.18–1.39	0.62	0.24
Induction alpha posterior	0.17–1.56	0.77	0.31	0.20–1.57	0.79	0.29
Rest theta frontal	0.32–.70	0.49	0.07	0.34–.69	0.50	0.07
Rest theta temporal-central	0.27–.70	0.49	0.08	0.29–.70	0.50	0.08
Rest theta posterior	0.18–.72	0.49	0.09	0.25–.71	0.51	0.08
Induction theta frontal	0.50–1.26	0.72	0.13	0.50–1.30	0.71	0.13
Induction theta temporal-central	0.50–1.20	0.70	0.13	0.51–1.25	0.69	0.13
Induction theta posterior	0.49–1.15	0.69	0.13	0.50–1.23	0.69	0.13

Values are log-transformed from power spectral density at alpha (8–13 Hz) and theta (4–8 Hz) frequency ranges.

#### Alpha

Consistent with the literature comparing alpha power during rest relative to active situations, a main effect of Task emerged (*F*(1, 197) = 104.86, *p* < 0.001, *η*^*2*^_*P*_ = 0.35), such that alpha power, collapsed across sites, was greater during rest (*M* = 0.70, *SD* = 0.25) than audio induction (*M* = 0.64, *SD* = 0.24, *t*(200) = 8.80, *p* < 0.001). Also in line with the well-established link between sleepiness and enhanced alpha power, this main effect was qualified by a significant Task X Sleepiness interaction (*F*(1, 197) = 15.29, *p* < 0.001, *η*^*2*^_*P*_ = 0.07), such that the difference in alpha power between rest and induction was smaller as induction sleepiness increased (*r* = -0.28, *p* < 0.001). There was also a main effect of ROI (*F*(1.31, 257.98) = 410.32, *p* < 0.001, *η*^*2*^_*P*_ = 0.68), such that irrespective of task, alpha power increased linearly from frontal to posterior regions of the scalp (*F*(1, 197)_lin_ = 440.81, *p* < 0.001, *η*^*2*^_*P*_ = 0.69; see Table 3 for mean values). These main effects were qualified by a significant Task X ROI interaction (*F*(1.49, 292.88) = 131.61, *p* < 0.001, *η*^*2*^_*P*_ = 0.40), such that the effect of ROI was greater during rest relative to audio induction (i.e., the magnitude of alpha power increase from frontal to posterior regions was larger during rest than audio induction; *F*(1, 197)_linXlin_ = 162.67, *p* < 0.001, *η*^*2*^_*P*_ = 0.45). Contrary to predictions, there were no significant interactions involving Group (*F*s < 0.39, *p*s > 0.589). Similarly, the independent-samples t-tests comparing alpha power between meditation and control audio without accounting for rest did not yield significant group differences across any region (*t*s <|.92|, *p*s > 0.361).

#### Theta

Again, consistent with a multitude of past studies showing increased theta during more cognitively demanding tasks relative to rest, an expected main effect of Task emerged (*F*(1, 197) = 1696.49, *p* < 0.001, *η*^*2*^_*P*_ = 0.90), such that theta power, collapsed across sites, was greater during the audio induction (*M* = 0.70, *SD* = 0.12) than rest (*M* = 0.50, *SD* = 0.08, *t*(200) = 41.65, *p* < 0.001). There was also a main effect of ROI (*F*(1.29, 253.71) = 14.77, *p* < 0.001, *η*^*2*^_*P*_ = 0.06), such that collapsing across task, theta power was greatest at the frontal (*M* = 0.60, *SD* = 0.10) region relative to both temporal-central (*M* = 0.59, *SD* = 0.10; *t*(200) = 4.60, *p* < 0.001) and posterior (*M* = 0.59, *SD* = 0.10; *t*(200) = 3.73, *p* < 0.001) regions; whereas theta power at temporal-central and posterior regions did not differ (*t*(200) = 1.27, *p* = 0.206). These main effects were qualified by a significant Task X ROI interaction (*F*(1.25, 252.41) = 53.64, *p* < 0.001, *η*^*2*^_*P*_ = 0.21), such that whereas theta power differed across all regions during the audio induction (*t*s >|3.02|, *p*s < 0.003; highest at the frontal region [*M* = 0.71, *SD* = 0.13], followed by temporal-central [*M* = 0.69, *SD* = 0.13], and posterior [*M* = 0.68, *SD* = 0.13], respectively), theta power did not differ by region during the rest task (*t*s <|1.27|, *p*s > 0.206). No other significant main or interactive effects emerged, including those involving Group (*F*s < 1.84, *p*s > 0.176). Independent samples t-tests comparing theta power between meditation and control audio without accounting for rest did not yield significant group differences across any region (*t*s <|.26|, *p*s > 0.797). Together, these results did not support the prediction that meditation would uniquely enhance theta power relative to controls.

### Prediction 3: effects of audio induction on LPP and NAS ratings

**Figure 2:**
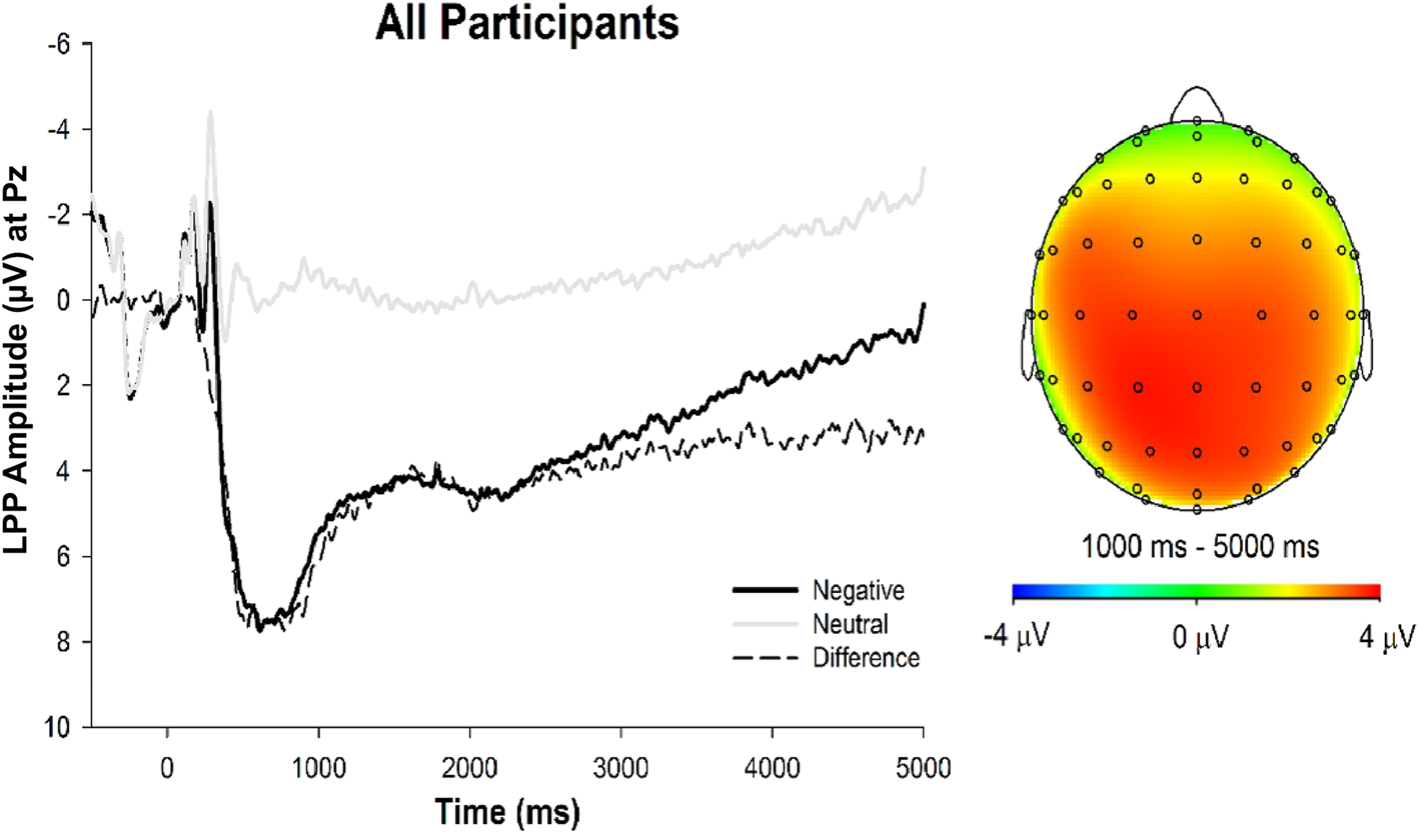
LPP waveforms and topographic head map of all participants. *Note.* Stimulus-locked grand average waveforms depicting the LPP (left). Grand average waveforms are computed by averaging each participant’s waveforms across negative (dark line) and neutral (grey line) trials at electrode site Pz, and then averaging across all participants. Time 0 represents the onset of the stimulus. Head map provides scalp topography of difference in response amplitude between negative and neutral trials across the 1,000–5,000 time ms window (right).

**Figure 3:**
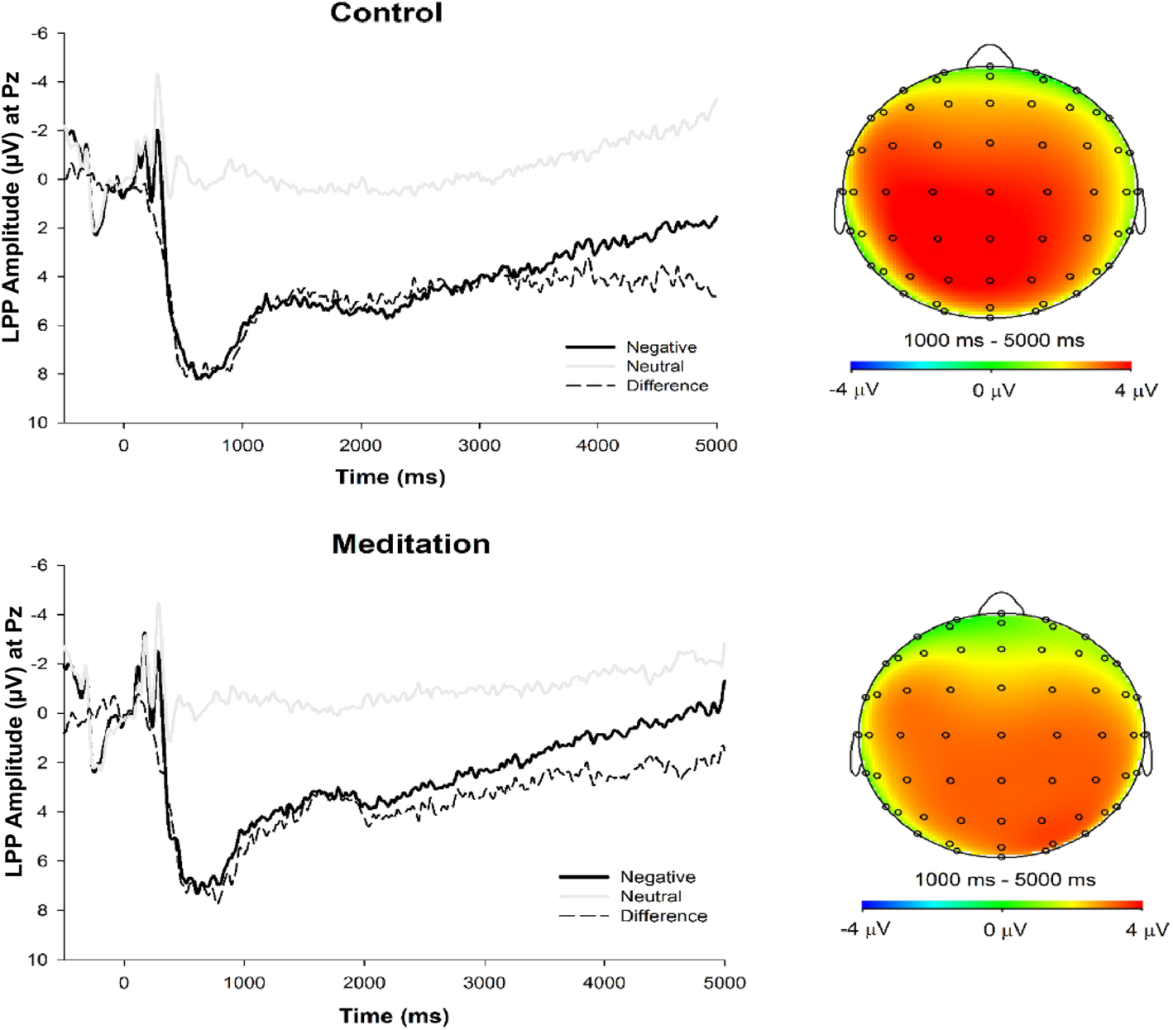
LPP waveforms and topographic head map by experimental group. *Note.* Stimulus-locked grand average waveforms depicting the LPP for control (top left) and meditation (bottom left) groups. Grand average waveforms are computed by averaging each participant’s waveforms across negative (dark line) and neutral (grey line) trials at electrode site Pz, and then averaging across group participants. Time 0 represents the onset of the stimulus. Head map provides scalp topography of difference in response amplitude between negative and neutral trials across the 1,000–5,000 ms time window.

Grand averaged ERP waveforms across all participants are presented in Fig. 2. ERP waveforms and amplitudes across the two experimental conditions are presented in Fig. 3 and Table 4, respectively.

**Table 4:** Means and SD for the Late Positive Potential (LPP) by group.

Valence by ERP window	Control N = 100			Meditation N = 101		
	*Range*	*M*	*SD*	*Range*	*M*	*SD*
Neutral 500–900 ms	− 19.65 to 17.95	− 0.03	5.51	− 12.84 to 15.73	− 0.35	5.74
Negative 500–900 ms	− 16.40 to 20.94	7.79	6.15	− 20.52 to 26.34	6.73	7.08
Neutral 1,000–2000 ms	− 16.21 to 18.48	0.23	5.43	− 13.00 to 16.04	− 0.33	5.30
Negative 1,000–2000 ms	− 15.13 to 15.72	5.27	5.95	− 13.22 to 23.43	3.73	7.02
Neutral 2000–3,000 ms	− 19.60 to 20.65	0.26	6.59	− 14.94 to 11.78	− 0.79	6.00
Negative 2000–3,000 ms	− 23.69 to 20.37	4.89	7.58	− 21.43 to 20.93	3.06	8.04
Neutral 3,000–4,000 ms	− 23.19 to 17.74	− 0.59	7.05	− 14.28 to 15.27	− 1.15	6.42
Negative 3,000–4,000 ms	− 23.92 to 20.32	3.45	8.17	− 20.14 to 21.01	1.56	8.15
Neutral 4,000–5,000 ms	− 25.06 to 18.04	− 1.97	7.81	− 26.41 to 14.74	− 1.92	7.28
Negative 4,000–5,000 ms	− 23.75 to 20.41	2.21	8.35	− 19.65 to 20.22	0.27	8.27
Neutral 1,000–5,000 ms	− 20.52 to 16.63	− 0.52	6.14	− 13.87 to 12.79	− 1.05	5.67
Negative 1,000–5,000 ms	− 21.62 to 18.33	3.95	7.08	− 16.42 to 18.64	2.16	7.50

Values are in microvolts (μV) extracted from electrode site Pz and time-locked to stimulus onset.

#### Early maximal LPP

As expected, a main effect of Valence emerged (*F*(1, 196) = 326.65, *p* < 0.001, *η*^*2*^_*P*_ = 0.63), such that negative stimuli elicited larger LPP amplitudes (*M* = 7.20, *SD* = 6.60) than neutral stimuli (*M* = -0.20, *SD* = 5.63). Consistent with Lin et al.^52^, there was no significant Valence X Group interaction (*F*(1, 196) = 1.06, *p* = 0.305, *η*^*2*^_*P*_ < 0.01) or any interactions involving Sleepiness (*F*s < 0.13, *p*s > 0.717).

#### Late sustained LPP

**Figure 4:**
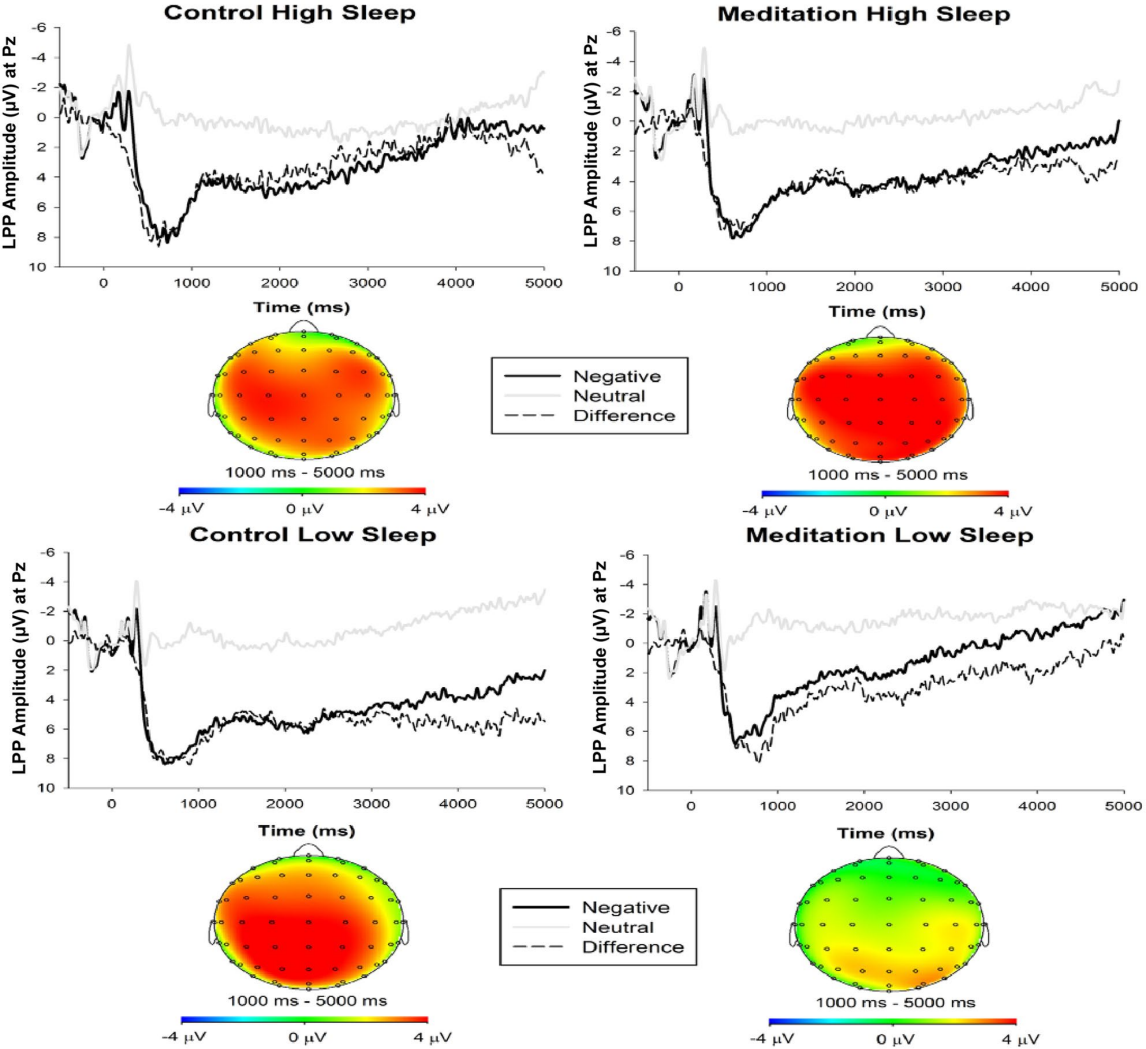
LPP waveforms and topographic head map by experimental group and sleepiness. *Note*. Stimulus-locked grand average waveforms depicting the LPP for control (left column) and meditation (right column) groups by high (top row) and low (bottom row) sleepiness separated by median split. Grand average waveforms are computed by averaging each participant’s waveforms across negative (dark line) and neutral (grey line) trials at electrode site Pz, and then averaging across group participants. Time 0 represents the onset of the stimulus. Head map provides scalp topography of difference in response amplitude between negative and neutral trials across the 1,000–5,000 ms time window. Less sleepiness during the guided meditation, but not control condition, was uniquely related to a smaller LPP on negative trials.

For the late sustained LPP, there were main effects of Valence (*F*(1, 196) = 32.73, *p* < 0.001, *η*^*2*^_*P*_ = 0.14) and Time (*F*(1.54, 301.65) = 47.17, *p* < 0.001, *η*^*2*^_*P*_ = 0.19), such that the LPP was more positive for negative stimuli, but reduced in positivity linearly over time irrespective of stimulus valence (*F*(1, 196)_lin_ = 57.61, *p* < 0.001, *η*^*2*^_*P*_ = 0.23). These main effects were qualified by a Time X Valence interaction (*F*(1.59, 311.19) = 6.94, *p* = 0.003, *η*^*2*^_*P*_ = 0.03), such that the difference in LPP amplitude by stimulus valence diminished linearly over time (*F*(1, 196)_linXlin_ = 8.55, *p* = 0.004, *η*^*2*^_*P*_ = 0.04). Notably, the main effect of Valence was also qualified by a three-way Valence X Group X Sleepiness interaction (*F*(1, 196) = 3.78, *p* = 0.053, *η*^*2*^_*P*_ = 0.02; see Fig. 4). No other significant interactions emerged (*F*s < 2.62, *p*s > 0.087).

To parse the three-way Valence X Group X Sleepiness interaction, Time was collapsed across Valence and the data was split by Group. To test whether the interaction between Sleepiness and Valence differed as a function of Group, follow-up correlational analyses involving Fisher r-to-z tests were conducted to compare the relationship between sleepiness and the LPP at each valence across groups. Analyses revealed a significant positive correlation between sleepiness ratings and the LPP on negative (*r* = 0.25, *p* = 0.014, 95% CI [0.05, 0.45]) but not neutral trials (*r* = 0.11, *p* = 0.290, 95% CI [-0.09, 0.31]) in the meditation group. In contrast, sleepiness ratings were unrelated to the LPP on neither neutral (*r* = 0.07, *p* = 0.473, 95% CI [-0.13, 0.27]) nor negative trials (*r* = -0.10, *p* = 0.313, 95% CI [-0.30, 0.10]) in the control group. Critically, the Fisher r-to-z tests yielded a significant group difference in only the correlation between sleepiness and the negative LPP (*z* =|2.45|, *p* = 0.014), but not the neutral LPP (*z* =|.24|, *p* = 0.81). Put more simply, less sleepiness during the guided meditation was uniquely related to the predicted smaller LPP on negative trials; see Fig. 4 for waveforms separated by group and sleepiness.

#### NAS ratings

The rANOVA yielded a main effect of Time (*F*(1, 206) = 135.41, *p* < 0.001, *η*^*2*^_*P*_ = 0.40), such that across the entire sample, NAS ratings increased from pre (*M* = 11.93, *SD* = 3.02) to post (*M* = 15.56, *SD* = 5.70, *t*(209) = 11.35, *p* < 0.001). Contrary to expectations, however, there were no significant interactions involving Group or Sleepiness (*F* < 3.29, *p*s > 0.07).

### Prediction 4: relationships between audio induction neural oscillatory activity and LPP

Contrary to predictions, meditative alpha, across all regions, was not related to either the early (*r*s < 0.16, *p*s > 0.122) or late sustained LPP (*r*s < 0.12, *p*s > 0.236). No significant correlations emerged in the control condition (early LPP: *r*s <|.15|, *p*s > 0.142; late sustained LPP: *r*s <|.07|, *p*s > 0.506).

Unexpectedly, meditative theta exhibited significant *positive* correlations with the early LPP across all three regions (frontal: *r* = 0.26, *p* = 0.010, 95% CI [0.07, 0.47]; temporal-central: *r* = 0.25, *p* = 0.013, 95% CI [0.06, 0.46]; posterior: *r* = 0.25, *p* = 0.015, 95% CI [0.05, 0.45]), such that higher meditative theta was associated with a larger negative-neutral difference in the early LPP response. Meditative theta was not however significantly correlated with the late sustained LPP across frontal (*r* = 0.19 *p* = 0.068, 95% CI [-0.01, 0.39]), temporal-central (*r* = 0.19, *p* = 0.062, 95% CI [-0.01, 0.39]), and posterior sites (*r* = 0.19, *p* = 0.062, 95% CI [-0.01, 0.39]). In contrast, no significant correlations emerged in the control condition for the early LPP (*r*s < 0.08, *p*s > 0.449) or late sustained LPP (*r*s < 0.13, *p*s > 0.230). Comparing correlations between groups using Fisher r-to-z transformations yielded trending but nonsignificant differences for the early LPP (frontal: *z* =|1.52|, *p* = 0.129, temporal-central: *z* =|1.32|, *p* = 0.187, posterior: *z* =|1.18|, *p* = 0.238), and no difference for the late sustained LPP (frontal: *z* =|.77|, *p* = 0.441, temporal-central: *z* =|.57|, *p* = 0.569, posterior: *z* =|.46|, *p* = 0.646) across the three regions. See Table 5 for full correlation tables. Although the directionality of the relationships was inconsistent with what was predicted, the observed correlations between meditative theta and the LPP nonetheless suggest that theta oscillatory activity may be a promising neural marker of OM meditation in predicting its subsequent effects on emotional reactivity.

**Table 5:** Partial correlations controlling for sleepiness among early LPP, late LPP, and change in neural oscillatory activity (audio induction minus rest) separated by group.

Control	1	2	3	4	5	6	7	8
1. Early LPP	–							
2. Late LPP	0.61**	–						
3. Alpha frontal	− 0.15	− 0.07	–					
4. Alpha temporal-central	− 0.09	− 0.03	0.86**	–				
5. Alpha posterior	− 0.02	0.05	0.69**	0.84**	–			
6. Theta frontal	0.05	0.08	0.34**	0.11	− 0.05	–		
7. Theta temporal-central	0.07	0.11	0.24*	0.11	− 0.02	0.89**	–	
8. Theta posterior	0.08	0.13	0.27**	0.19	0.13	0.75**	0.92**	–

Meditation	1	2	3	4	5	6	7	8
1. Early LPP	–							
2. Late LPP	0.73**	–						
3. Alpha frontal	0.16	0.12	–					
4. Alpha temporal-central	0.06	0.05	0.82**	–				
5. Alpha posterior	0.03	0.02	0.74**	0.88**	–			
6. Theta frontal	0.26*	0.19	0.40**	0.28**	0.14	–		
7. Theta temporal-central	0.25*	0.19	0.28**	0.26**	0.14	0.92**	–	
8. Theta posterior	0.25*	0.19	0.27**	0.29**	0.19	0.85**	0.96**	–

Early LPP and late LPP quantified as the average difference in response amplitude between negative and neutral trials across the 500–900 and 1,000–5,000 time ms windows, respectively; Alpha and theta values computed as difference in log-transformed power spectral density between audio induction and rest (* p < 0.05, ** p < 0.01).

## Discussion

The present study sought to advance understanding of the emotion regulatory effects of mindfulness meditation. Specifically, it aimed to discern whether brain changes occurring during meditation reflect mechanisms through which mindfulness meditation confers its “off-the-cushion” benefits to emotion regulation. Toward this end, the study leveraged a multimodal experimental approach adapted from Lin et al.^52^ to test four predictions: (1) there would be no baseline group differences in trait and state measures and that participant responses on the manipulation check would replicate Lin et al.^52^; (2) participants who engaged in guided mindfulness meditation would uniquely exhibit increased alpha and theta power during meditation relative to rest; (3) meditation would produce less emotional reactivity (LPP) and self-reported negative affect (NAS ratings); and (4) the predicted changes in alpha and theta power during meditation would correlate with the predicted reductions in the LPP. Critically, results revealed mixed support for these respective predictions insofar that: (1) with the key exception of sleepiness during the audio manipulation, there were no notable group differences in any of the baseline trait and state measures or manipulation check responses; (2) there were no group differences in alpha or theta power; (3) self-reported sleepiness during the audio manipulation moderated the predicted effect of meditation in reducing LPP amplitude, but NAS ratings did not differ by group; (4) increased theta power, but not alpha power, between meditation and rest was related to larger LPP amplitudes. Collectively, our findings provide an empirical demonstration that subjective states of vigilance (i.e., sleepiness) and neural activity (i.e., theta power) during the actual practice of OM meditation may influence its subsequent “off-the-cushion” effects on emotional reactivity in one of the largest novice non-meditator samples to date.

### Main analyses and predicted outcomes

#### Neural oscillatory activity during OM meditation

Unexpectedly, the guided meditation did not produce demonstrable differences in alpha or theta power relative to the control audio even after accounting for sleepiness. This null finding is inconsistent with previous suggestions that mindfulness meditation produces increased alpha and theta power relative to rest^58,111^. The absence of this effect in the current study signals the need for careful evaluation regarding the boundary conditions undergirding the ability to detect the spectral power effects reported in the mindfulness literature. Toward this end, three prescriptive factors long noted in critical reviews of contemplative science appear pertinent to consider: meditation experience, training duration, and meditative technique^11,12,35,112,113^.

Indeed, the ability to detect change in meditative neural oscillatory activity may depend on the meditation experience of the sample, and or the frequency and duration of meditative training. Therefore, one parsimonious consideration is that the design parameters of the study (i.e., one 20-min session of meditation with novice participants) may have been insufficient in both frequency and duration to produce group-level differences in alpha and theta power. This possibility is in line with Tang et al.^111^ recent proposal that meditative alpha and theta activity are moderated by progressive stages of mastery, where the early stage (of which our sample undoubtedly falls) involves minimal alpha and theta activity but is instead predominated by beta activity reflective of mind wandering and effortful control. Alpha and theta synchronization is then thought to accompany the transition to later stages, as practitioners strengthen concentration and expend less effort to maintain internal awareness.

Along these lines, a study-by-study reexamination of Lomas et al.^58^ systematic review revealed that only three of twelve studies reporting increased meditative alpha^103,104,114^ and three of fourteen studies reporting increased meditative theta^103,105,115^ were comprised of novices completing a single session of meditation; the rest involved advanced practitioners or weeks-long training intervals. Although this pattern does not demonstrate that mindfulness meditation is unrelated to alpha and theta power in novice meditators, it nonetheless constitutes a valuable observation to inform future work. It also must be noted that very few studies to date have compared within-subject changes in meditative oscillatory activity to an active control group (none of the novice studies cited above employed controls; but see Baijal and Srinivasan^116^ for a controlled study involving experienced meditators), challenging the extent to which reported changes reflect unique properties of meditation or, as the current findings suggest, reflect non-meditative components of the task (e.g., listening to an instructive audio with eyes closed). Indeed, without a control group, we likely would have interpreted our data as a demonstration of an effect of meditation. Consequently, we strongly advocate the incorporation of active control conditions in future studies as a foundational step toward parsing apart these competing possibilities.

Moreover, nuanced but potentially meaningful variation in meditation technique may differentially influence meditative alpha and theta power. Strikingly, all but *one* of the aforementioned studies reporting increased meditative alpha and theta in novice practitioners involved a variant of breath focused meditation^103–105,114^. Explicit instructions to monitor the breath via counting or biofeedback manipulation, as opposed to open monitoring of arising experience (as instructed in the present study) may preferentially recruit performance monitoring of sustained attention to a preselected target object^117^—a process that is known to elicit alpha and theta synchronization^59,118^. Because breath focus is conceptualized as a form of FA meditation^6,119^, our null finding is a reminder that although FA and OM meditation are often subsumed under the term mindfulness meditation, each may involve unique neural and functional properties^88,117,120,121^. Therefore, it may be prudent to carefully consider these distinctions during the design of future mindfulness meditation studies. Along these lines, a potentially fruitful follow-up study is to directly compare alpha and theta power during FA and OM meditation in novices.

In sum, our findings suggest that the functional significance of meditative alpha and theta is rife with nuance and in need of further clarification. In carrying out this work, it is important to recognize the general challenge of mapping brain measures to phenomenological states. For example, studies have reported increased alpha and theta power during states of mind wandering^122–125^, thereby challenging unilateral interpretations of alpha and theta synchronization as markers of internally-directed concentrated attention. In a convincing demonstration of this point, Brandmeyer and Delorme^126^ found that novice meditators did not show dissociable levels of theta power between meditation and mind wandering, whereas advanced practitioners exhibited enhanced theta power during meditation relative to mind wandering. To begin addressing these issues, EEG machine-learning models appear to hold substantial promise toward the development of more refined and precise measures of subjective mental states^125,127^.

#### Effect of OM meditation on the LPP: the moderating role of sleepiness

In analyzing the baseline and manipulation check measures prior to testing the predictions involving the effect of OM meditation, we found an unexpected difference in sleepiness during the audio induction (not measured in Lin et al.^52^), such that participants reported more sleepiness during the guided meditation than the control audio. Importantly, sleepiness differed only during the audio induction but not during rest and the picture viewing task, suggesting that the guided meditation may have *induced* sleepiness and counters the possibility of a baseline group difference in overall sleepiness. Given the substantial influence of sleepiness on a range of neurobiological functions^128–131^, accounting for sleepiness in meditation research (particularly in novice samples) may help explain some of the variability within the literature.

Directly supporting this suggestion, we found that sleepiness during the guided meditation moderated the expected effects on the late LPP but not the early LPP. The absence of early LPP modulation replicated Lin et al.^52^ and is consistent with another brief mindfulness induction study (Eddy et al.^107^). The current finding strongly supports the prevailing notion that a single-session mindfulness induction is insufficient to modulate bottom-up attentional mechanisms during early emotion processing^42,52,107,132^. The question remains, however, whether prolonged OM meditation training can modulate the early LPP. Although cross-sectional studies involving experienced meditators report smaller early LPP responses relative to novices^50,133^, longitudinal studies are needed to corroborate the assumption that extensive mindfulness training reduces the early LPP.

Expanding upon the prediction that the guided meditation would attenuate the late sustained LPP, the current study found that sleepiness during meditation moderated the effect of meditation on the late LPP. Critically, participants low on sleepiness exhibited a smaller difference between negative and neutral LPP amplitudes, whereas participants high on sleepiness exhibited a sustained difference between negative and neutral LPPs similar to the pattern demonstrated in controls. Notably, this effect was driven by a positive correlation between sleepiness and negative trials (i.e., less sleepiness, smaller negative LPP amplitude), suggesting that the guided meditation reduced the negative-neutral LPP difference in non-sleepy participants through attenuating the response to negative pictures. Interpretatively, the emotion regulatory effect of OM meditation appears contingent on whether participants were sufficiently awake and alert to participate in the guided meditation. Moreover, these findings help rule out the otherwise theoretically tenable possibility that the results of Lin et al.^52^ were driven by sleepiness *from* the meditation rather than the meditation itself. Indeed, we view this as an empirical demonstration of past suggestions^94^ that a first-time exposure to OM meditation can induce sleepiness in novice practitioners. Moreover, our findings expand these considerations by showing that sleepiness during meditation can systematically influence dependent outcome measures and therefore should be adequately accounted for in future meditation studies.

Despite promising implications, much remains unknown about the underlying mechanisms of mindfulness-related LPP attenuation. Conservatively speaking, it is rather remarkable that this effect was evidenced in the first place. First, modulation of the LPP is unlikely to occur by chance. In fact, sustained difference in the LPP response between negative and neutral stimuli have been shown to persist over time, even after repeated exposure^134,135^. Therefore, that novice non-meditators, after completing a brief session of OM meditation, would exhibit any measurable change in emotion processing is notable to say the least. With that said, our findings are in line with a growing number of electrophysiological studies reporting unique effects of brief mindfulness exercises on a wide range of psychological functions in novice samples^52,85,136–140^, raising more questions than answers regarding the theoretical and empirical boundary conditions distinguishing brief exposure to mindfulness training from an established meditation practice. Second, whereas many studies have demonstrated LPP reduction in response to antecedent-focused viewing strategies (e.g., cognitive reappraisal; see Hajcak et al.^141^, for a review), the current findings replicate Lin et al.^52^ in that LPP attenuation was observed without any explicit viewing instruction and supports the developing idea that mindfulness meditation engenders implicit non-voluntary down-regulation of emotional arousal.

Indeed, these considerations echo the growing speculation that mindful emotion regulation may involve distinct neural mechanisms separable from voluntary “top-down” strategies^7,39^. In this vein, Uusberg and colleagues’^142^ elegant demonstration of affective adaptation as a function of state mindfulness provides a compelling mechanistic framework—namely that mindfulness initially produces an increase in the LPP response followed by a marked reduction. Despite the key caveat that Uusberg et al.^142^ findings were derived from a state mindfulness induction (i.e., participants were instructed to view pictures mindfully over repeated trials), a similar mechanism may extend to the present study insofar that the OM meditation may have promoted affective adaptation as participants were progressively exposed to more negative high arousing stimuli.

#### Self-reported affect

Lastly, NAS ratings increased from pre- to post-experiment across the whole sample. Contrary to predictions, there were no group differences in post NAS ratings or the pre-post difference. The global increase in NAS ratings likely reflected the design of the study, such that participants were instructed to rate their affect after exposure to a series of highly negative arousing images that are known to induce transient negative affect^143^. Parsimoniously, these findings suggest that the guided meditation did not modulate self-reported negative affect. However, it is also possible that the NAS lacked the sensitivity to detect group differences. Confounding variables (e.g., physical discomfort, boredom) related to the study procedures and lengthy duration between measurements (NAS was measured before and after completion of the rest, audio induction, and picture viewing tasks) may have masked or superseded the effect of the experimental manipulation. Furthermore, the NAS is an aggregate metric comprised of a broad range of feelings such as guilt, hostility, and irritability that may be less contextually relevant to picture viewing. Put more simply, it was unclear whether the global increase in NAS ratings reflect a response to picture viewing (what we intended to measure) or the “ancillary” distress associated with the demands and length of the session more broadly.

#### Relationships between neural oscillatory activity during meditation and LPP

In examining the relationship between meditative neural oscillatory activity and the LPP, we found that meditative theta, but not alpha, was moderately and marginally correlated with the early and late sustained LPP, respectively. Specifically, participants with higher meditative theta, across all three scalp regions, exhibited larger LPP responses. Comparing the correlations across group revealed trending differences for the early LPP but not the late sustained LPP. Despite the lack of statistical significance, the fact that the analyses were preplanned, coupled with the observation that the magnitude of the correlations were remarkably stable across all scalp regions, suggests that the relationship between meditative theta and the early LPP is at least worth elaboration.

Curiously, the directionality of the relationship is surprising and seemingly inconsistent with the extant literature. Given that mindfulness meditation has been associated with increased theta power and reduced emotional reactivity, it stood to reason that enhanced meditative theta would relate to a reduced, *not* enhanced LPP response. The current finding, however, suggests that this line of reasoning needs revision. As previously discussed, studies involving novice practitioners reporting enhanced meditative theta have primarily utilized breath-oriented FA meditations. The reported theta synchronization during FA meditation may reflect the recruitment of sustained attention and cognitive control during counting or active breath monitoring^118,144^. In this light, enhancement of theta power during FA meditation may functionally reflect the cultivation of concentration, rather than the fostering of nonreactive awareness typically associated with OM meditation^6^. Consequently, because the current study utilized an OM meditation and did not observe a unique increase in meditative theta (over and above that of the control condition), the functional significance of meditative theta as an individual difference measure is unclear.

Because enhanced meditative theta and alpha are most often and consistently observed in advanced meditators rather than novices^57,58^, their respective relationship to theoretically relevant constructs may too vary as a function of meditative experience. Researchers have long cautioned that similar or even identical measures may reflect different latent constructs and or underlying neural processes depending on the meditation expertise of the sample^11,12,34,145,146^. It appears plausible that the relationships between meditative neural activity (i.e., what is occurring during meditation) and abilities cultivated through meditation itself (i.e., what is being trained during meditation) would change with extended meditation training. This possibility is consistent with Cahn and Polichs’^57^ conclusion that state changes in meditative oscillatory activity is likely to manifest as trait level changes with continued practice.

## Conclusion

In summary, the predictions received mixed support with notable qualifications: (1) the guided meditation did not produce demonstrable differences in alpha and theta power relative to controls; (2) sleepiness during the guided meditation moderated the effect of meditation on the late LPP, such that less sleepiness corresponded to a smaller difference between negative and neutral LPP amplitudes; (3) NAS ratings did not differ between groups; (4) meditative theta, but not meditative alpha, was positively correlated with the early LPP, but did not statistically differ relative to controls. Taken together, the findings call into question whether extant functional interpretations of meditative oscillatory activity are applicable to novice practitioners^58^, and shed light on the conditions under which brief mindfulness meditation modulates emotion processing. This systematic extension of Lin et al.^52^ demonstrates not only the value of large sample replication, but also highlights the need for incremental research to test previous claims and unexamined assumptions. Equally important, the current study exemplifies the unique challenge of conducting contemplative science research—namely, that meditation specific factors (e.g., meditation experience, meditative style, training duration, etc.) can interact with broader issues concerning operationalization and measurement to complicate the development of sound predictions and generalizable conclusions.

Echoing Van Dam et al.^12^ sentiments, it would appear that the public media is not the only avenue through which the benefits of mindfulness meditation are susceptible to exaggeration. Conceptual and methodological challenges render scientific investigations equally susceptible to the drawing of premature and likewise exaggerated conclusions. The validity of the neural correlates and putative neural mechanisms of mindfulness meditation appears contingent on a host of conceptual, methodological, and sample dependent factors. Given the constellation of mixed and qualified findings from the current study, it must be acknowledged that contrary to past suggestions, any effect derived from a single brief mindfulness meditation observed in novice non-meditators is likely dependent on a range of contextual factors including the definition and operationalization of the term “mindfulness meditation” itself. Furthermore, it remains unclear how meditation related findings from a controlled laboratory setting would generalize to daily life. In this light, efforts aimed at developing any unitary or holistic framework to explain how mindfulness meditation works appear premature. Perhaps a more tractable and practical endeavor involves identifying and understanding the contextual factors that undergird the ways in which mindfulness meditation has been operationalized and previously shown to “work”.

Amidst the humbling complexity and mounting skepticism, the current findings inspire new directions toward understanding the effects of brief mindfulness meditation in novice practitioners. Specifically, it may be prudent to add sleepiness to the growing list of prescriptive factors for contemplative science—further exploration of its role may help clarify variability in the extant literature and inform the development of future studies. Given that sleepiness is known to modulate attention and emotion processing^128,130^, past studies, particularly those involving meditation novices and brief interventions, may benefit from replication efforts that include measures of sleepiness. Furthermore, the discovery that meditative theta related to the early LPP sparks novel and potentially fruitful lines of inquiry. If replicated, the unique directionality of the relationship may lead to valuable insights about the functional significance of meditative theta in novice practitioners. Moreover, given that the LPP is a well-established ERP measure of emotion processing, elucidating the nature of its relationship to meditative theta could be valuable for future studies of mindful emotion regulation. Examining whether meditative theta is sensitive to change as a function of prolonged OM meditation training may be a foundational step in establishing a neural index of meditation quality (i.e., a measure that is sensitive to changes that occur acutely during a single session of meditation *and* over the course of longer-term meditation practice). Toward this end, employment of cutting-edge time-frequency^147^, source mapping^148^, and machine-learning^125,127^ approaches appear particularly promising. Furthermore, it must be acknowledged that the current study involved an all-female sample, and therefore it is unknown to what extent the aforementioned considerations generalize to males. Given the recent proliferation of studies highlighting gender differences in mindfulness research^77–79,149^, including the very pertinent finding that males are underrepresented in mindfulness studies^150^, we strongly encourage future replication efforts with a large balanced sample.

Although the current study yielded more questions than answers, it did not diminish the intrigue, challenge, and promise in linking what occurs during meditation with its “off-the-cushion” effects. Given the vast history, rich intricacy, and prevailing mystery of contemplative practice, there is perhaps no reason to expect anything less.

## Data Availability

The dataset is available from the corresponding author upon reasonable request.
